# Biological poisons targeting the pituitary gland: insights across the five kingdoms

**DOI:** 10.3389/fendo.2025.1708792

**Published:** 2025-11-13

**Authors:** David Sanchis-Pascual, Rosa Cámara Gómez, Pilar Morillas-Amat, Darío Lara-Gálvez, Víctor Pérez-Cervantes, Pilar Masdeu-López-Cerón, Juan Francisco Merino-Torres

**Affiliations:** 1 Endocrinology and Nutrition Department, University and Polytechnic Hospital La Fe, Valencia, Spain; 2 Joint Research Unit on Endocrinology, Nutrition and Clinical Dietetics, Health Research Institute La Fe, Valencia, Spain; 3 Pediatrics Department, Primary Health Care Center of Silla, Silla, Spain; 4 Medicine Department, University of Valencia, Valencia, Spain

**Keywords:** pituitary gland, poison, toxin, venom, nature

## Abstract

The pituitary gland, as a central regulator of endocrine function, may be affected by a wide range of biologically derived harmful substances present in nature. While most available literature focuses on neoplasms, trauma or autoimmune disorders, the potential impact of natural toxins and poisons on pituitary function remains underexplored. This narrative review addresses the effects of acute or chronic exposure to harmful agents originating from the five biological kingdoms—Animalia, Plantae, Fungi, Monera, and Protista—on the hypothalamic–pituitary axis. Drawing on clinical reports, experimental data, and physiological insights, we describe how various biological substances may alter hormonal regulation, leading to temporary or persistent dysfunction. Importantly, this review does not cover direct infectious involvement of the pituitary, such as pituitary abscess, but focuses instead on biologically active compounds produced by living organisms. The review highlights a dispersed body of knowledge with implications not only for endocrinologists and pituitary specialists, but also for clinicians in toxicology, emergency medicine, infectious diseases, and global health. Recognizing the pituitary as a vulnerable target in diverse environmental and ecological contexts may aid in the early diagnosis and management of otherwise unexpected endocrine disorders.

## 1 Introduction

The pituitary gland is a pivotal endocrine structure situated at the base of the brain, within the sella turcica, and connected to the hypothalamus via the pituitary stalk (1). Despite its small size, it plays a central role in regulating the entire endocrine system through its anterior and posterior lobes. The anterior pituitary (adenohypophysis) secretes trophic hormones and prolactin, that govern target organs such as the adrenal glands, thyroid, gonads, and liver (2). The posterior pituitary (neurohypophysis), derived from neural tissue, releases oxytocin and vasopressin in response to hypothalamic input (3). This bidirectional communication is tightly regulated by negative feedback loops, allowing for dynamic adaptation to internal and external stimuli such as stress, fasting, circadian rhythms, and physiological demands (4, 5). Pituitary integrity is therefore essential to homeostasis, and its disruption may result in substantial clinical consequences, including hormone deficiencies, metabolic dysregulation, and impaired stress responses (6–8).

Although the pituitary operates under robust physiological control, various insults may compromise its function or structure (9, 10). These insults may lead to partial or complete hypopituitarism, hormone hypersecretion, or mass effects due to glandular inflammation, hemorrhage, or expansion. Beyond endocrine dysregulation, hypopituitarism is associated with increased clinical risk. Mortality rates among patients with hypopituitarism are higher than those in the general population, with a meta-analysis reporting a standardized mortality ratio of 1.55 (95% CI 1.14–2.11), even after hormone replacement therapy (11, 12). Furthermore, they also show increased rates of ICU admission and longer hospital stays compared to individuals without pituitary dysfunction (13). These findings underscore the importance of early recognition and prevention of pituitary injury. It is therefore essential to understand how the pituitary gland can be damaged in various pathological contexts, as several causes such as mass effects, radiation, trauma, infections, autoimmune processes, ischemia, and infiltrative diseases have been described (14–20).

While these mechanisms are well established, the potential for naturally occurring biologically derived compounds—such as venoms, plant poisons, fungal toxins, and microbial exotoxins—to impair pituitary function remains underrecognized. This review addresses their impact across the five biological kingdoms, emphasizing an underexplored intersection between natural toxins and endocrine regulation, emphasizing its clinical relevance for recognizing pituitary dysfunction in toxic exposures where early hormonal screening may be crucial for diagnosis and management.

This work was conducted as a narrative review based on an exploratory search of the biomedical literature in PubMed and Google Scholar. Iterative combinations of keywords were used, including *“pituitary”* together with *“toxin,” “venom,” “poison,” “endocrine,”* and terms referring to biological sources such as animals, plants, fungi, bacteria, and protists. Additional references were identified from the bibliographies of selected papers. No formal time or language restrictions were applied, and relevant publications from the 1980s to June 2025 were considered.

## 2 Biological kingdoms and pituitary dysfunction

**Figure 1:**
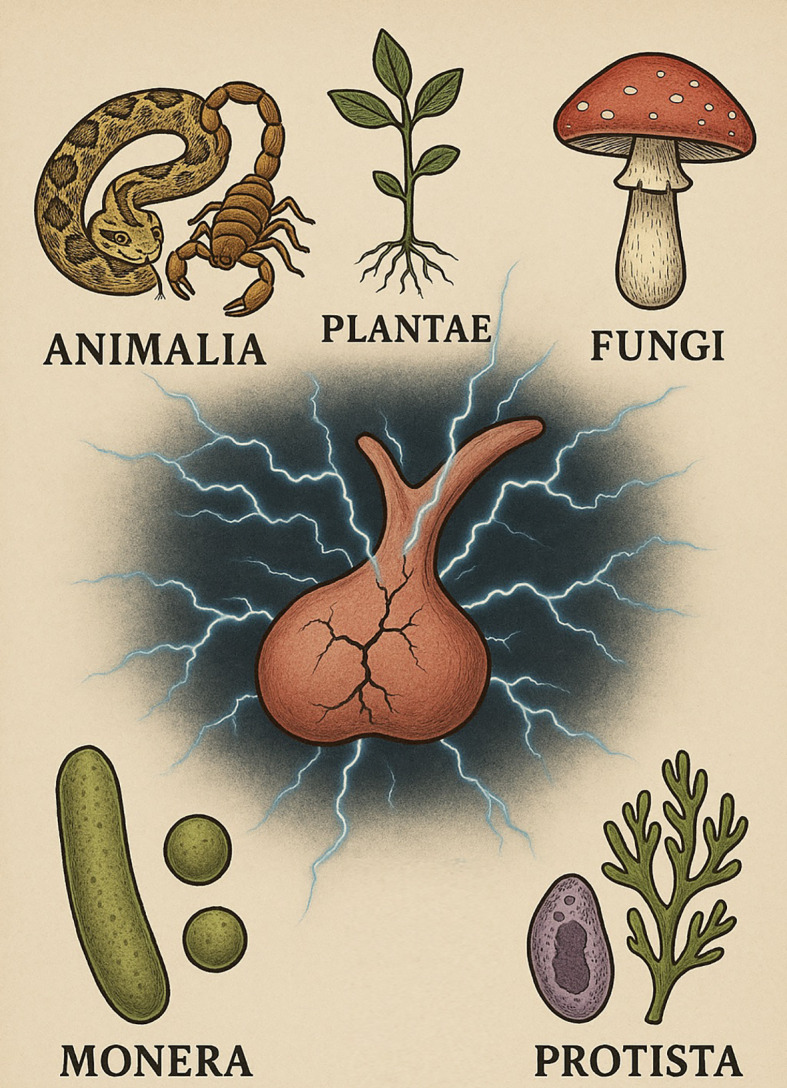
Schematic representation of the five biological kingdoms (Animalia, Plantae, Fungi, Monera and Protista) and their potential impact on pituitary function. AI-assisted visualization; verified by authors.

In this review, we adopt the classical five-kingdom model of biological classification proposed by *Whittaker* in 1969 as a conceptual framework to explore naturally occurring agents capable of injuring the pituitary gland (**Figure 1**) (21). Although more recent taxonomic systems such as the proposal by Cavalier-Smith, which incorporates *Chromista* and other eukaryotic groups, the five-kingdom scheme remains widely used in educational and medical literature and offers a practical structure for grouping biologically derived toxins (22). These kingdoms encompass a broad spectrum of organisms capable of producing venoms, poisons, or other biologically derived compounds that may affect pituitary function, either directly or indirectly.

### 2.1 Kingdom *Animalia*

The kingdom *Animalia* comprises an immense variety of multicellular species, ranging from complex vertebrates to microscopic invertebrates, many of which have evolved specialized biochemical defenses or predatory tools in the form of venoms and toxins (23). These bioactive compounds often target critical physiological processes in prey or predators, such as the cardiovascular and nervous systems (24, 25). The endocrine system, and particularly the pituitary gland, can also be affected, most often in the context of snake envenomation, but also by venom components from other animal groups such as arachnids and insects, through mechanisms ranging from direct neuroendocrine interference to indirect vascular and inflammatory injury (26–28).

#### 2.1.1 Snake envenomation

Snake envenomation is a major public health issue in tropical Asia, causing 1.8–2.7 million cases and 81,000–138,000 deaths annually, mostly in India (29, 30). It is a multisystemic condition involving renal failure, coagulopathy, myotoxicity, cardiac and neurological complications (31). Endocrine complications have also been described, most notably pituitary dysfunction, although adrenal and thyroid involvement have occasionally been reported (26, 32, 33).

The association between snakebite and hypopituitarism was first recognized in 1958, when Wolff reported a case following *Bothrops jararacussu* envenomation (34). However, the majority of clinical evidence originates from envenoming by Russell’s viper (*Daboia russelii* and *D. siamensis*), which has become the paradigm species linking snake venom to pituitary failure (35–39). Although most reports concern anterior pituitary dysfunction, involvement of the neurohypophysis has also been described, including cases of ADH deficiency (40–42).

**Figure 2:**
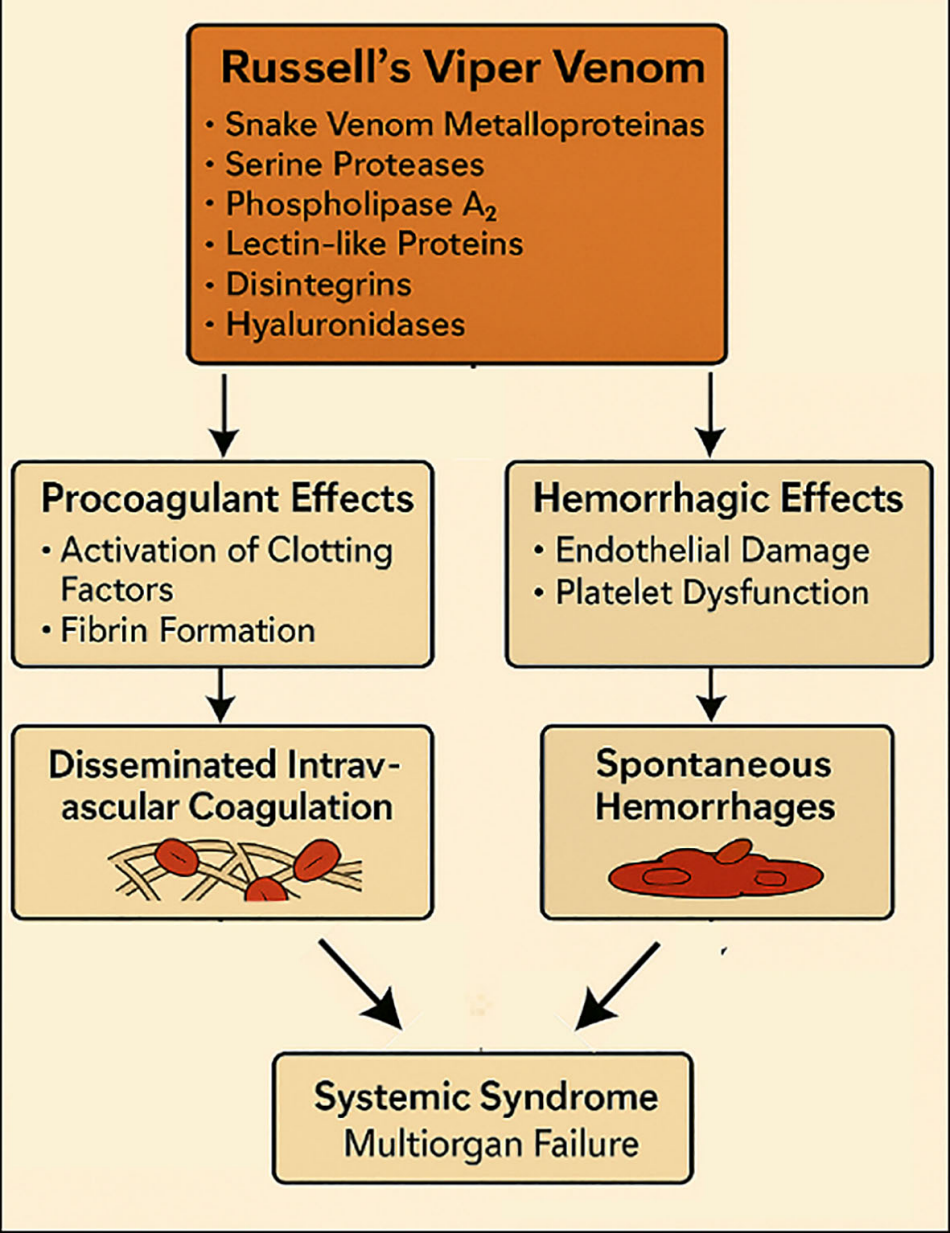
Mechanisms of Russell’s viper venom, including coagulopathy, endothelial injury, and disseminated intravascular coagulation leading to multiorgan failure. AI-assisted visualization; verified by authors.

Russell’s viper venom is a complex mixture of bioactive molecules, notably snake venom metalloproteinases, serine proteases, phospholipase A_2_, lectin-like proteins, disintegrins, and hyaluronidases (43, 44). These enzymes act synergistically on hemostasis, directly activating clotting factors V and X, driving rapid thrombin generation and fibrin formation, while thrombin-like enzymes accelerate fibrinogen consumption and fibrinolysis (**Figure 2**) (45). Concomitantly, metalloproteinases (“hemorrhagins”) damage vascular endothelium, and PLA_2_ and lectin-like proteins impair platelet function, further amplifying the coagulopathy (46, 47). The net effect is a state of profound endothelial injury with disseminated intravascular coagulation and spontaneous hemorrhages (48).

Clinically, two forms are recognized: acute and chronic hypopituitarism (35). Diagnosis of the acute form is challenging because glucocorticoid therapy and non-thyroidal illness can mask hormonal deficits. Nevertheless, *Tun-Pe* et al. identified combined deficiencies of cortisol, prolactin, and growth hormone (GH) in nine patients with altered consciousness and hemodynamic instability after Russell’s viper envenomation (35). Similarly, *Proby* et al. described multiple hormonal deficits in patients with snakebite-associated acute kidney injury, with 10 of 15 exhibiting cortisol deficiency, 19 of 20 reduced thyroid stimulating hormone (TSH) and T4, and 12 of 17 men showing low testosterone with inappropriately normal gonadotropins (49). Although methodological limitations exist, the persistence of deficits in 11 of 12 patients argues against transient illness-related changes and supports true pituitary damage. *Rajagopala* et al. further reported that acute hypopituitarism typically emerges around 9 days after envenomation, with unexplained hypoglycemia (100%) and refractory hypotension (67%) as key clinical markers (36). Autopsy studies have revealed focal pituitary hemorrhages and fibrin thrombi consistent with ischemic and hemorrhagic injury, while other work has suggested a paradoxical early hypersecretion of TSH, GH, and adrenocorticotropic hormone (ACTH) within the first hours, probably reflecting toxin-induced stimulation rather than destruction (50, 51).

Chronic hypopituitarism is typically diagnosed years after the initial bite, with a mean delay of 8.1 ± 3.6 years (37). The nonspecific nature of symptoms—including fatigue, hypothyroidism, hypogonadism, and adrenal insufficiency—often accounts for diagnostic delay. Central hypothyroidism and gonadotropin deficiency are almost universal, with ACTH deficiency present in approximately 93% of cases (38). GH deficiency shows wider variability, ranging from 15% in the cohort studied by *Bhat* et al. to 100% in that of *Naik* et al. (39, 52) Radiological changes are frequent, as *Ayan Roy* et al. reported magnetic resonance imaging abnormalities in all patients studied (n=15), with partial (47%) or complete (53%) empty sella (38). Furthermore, stalk thinning and proximal T1 hyperintensity was identified in 23% and 15% respectively.

Predictors of pituitary dysfunction remain controversial. *Naik* et al. found no clear differentiating factors between patients with and without hypopituitarism (39). In contrast, *Bhat* et al. identified coagulopathy and the need for hemodialysis as predictors in patients with acute kidney injury, while *Rajagopala* et al. associated multiorgan failure, thrombocytopenia, and transfusion requirements with the later development of pituitary insufficiency (36, 39, 52). These endocrine alterations likely reflect a complex combination of direct ischemic–hemorrhagic injury to the gland and systemic inflammatory stress associated with coagulopathy, rather than a purely toxin-mediated pituitary effect.

Pituitary involvement has also been described beyond Russell’s viper. In fact, a case report of *Gloydius blomhoffii* envenomation documented transient secondary adrenal insufficiency presenting with severe hyponatremia, low cortisol, and inadequate responses to dynamic stimulation tests, in the context of a pre-existing empty sella (53).Furthermore, experimental work in rabbits showed that the neurotoxic fraction of *Naja haje* (Egyptian cobra) venom generates an hyperglycemic status, as evidenced by a paradoxical reduction in pituitary ACTH secretion alongside elevated circulating cortisol (54). Another noteworthy example is the green mamba (*Dendroaspis angusticeps*) venom, from which the peptide mambaquaretin (MQ1) has been isolated (55). Although it does not target the pituitary directly, this venom-derived peptide MQ1 is a highly selective vasopressin V2 receptor competitive antagonist, blocking β-arrestin and MAP kinase (MAPK) pathways, and *in vivo* producing a pure aquaretic effect, with potential therapeutic applications in disorders of vasopressin signaling such as polycystic kidney disease or inappropriate ADH secretion.

#### 2.1.2 Other venomous species

Beyond reptiles, evidence from the phylum Arthropoda—particularly arachnids and insects—also points to the modulation of pituitary function (56). *Daachi* et al. reported that *Androctonus australis hector* scorpion venom increased ACTH and corticosterone levels and disrupted circadian rhythmicity. Histological analysis revealed enhanced ACTH immunoexpression in the anterior pituitary, together with increased oxidative markers and greater vascular permeability in the hypothalamus (57). In addition, venoms from species such as *Leiurus quinquestriatus* var. *hebraeus* and *Buthus martensii* have been used in more mechanistic studies of pituitary physiology, the former to investigate regulatory feedback in ACTH secretion and the latter to explore sodium current–dependent membrane potentials in anterior pituitary cells (27, 58). Moreover, α-latrotoxin, a component of black widow spider venom, has been shown to trigger vasopressin and oxytocin release through its interaction with calcium-independent receptors localized in the plasma membrane as well as calcium-dependent receptors for latrotoxin (59). Regarding insects, the available evidence is more limited but nonetheless illustrative. Sapeptin B, an antibacterial peptide from the flesh fly, has been shown to inhibit potassium currents in GH3 pituitary cells, while mastoparan, a wasp venom peptide, stimulates prolactin secretion in rats through increased intracellular calcium (28, 60). Finally, bee venom has been reported to deplete secretory granules in corticotrophs and somatotrophs, accompanied by elevated plasma ACTH and GH concentrations. These effects have led some authors to propose bee venom as a potential enhancer of growth performance and a modulator of pubertal timing in certain mammalian species (61, 62).

In these species, the observed hormonal changes are thought to result primarily from neuroendocrine stress responses rather than direct pituitary cytotoxicity, as no histological lesions or direct cellular injury have been demonstrated, supporting a predominantly neuroendocrine or systemic stress mechanism.

### 2.2 Kingdom *Plantae*

Kingdom *Plantae* is characterized by multicellular, autotrophic organisms with cell walls of cellulose and photosynthetic capacity (63). Although plants play a role in numerous human physiological functions and are widely used for their therapeutic potential, some plant-derived compounds can also exert harmful effects (64, 65). However, their influence on the hypothalamic–pituitary axis remains poorly understood. This section focuses on the impact of whole plants or natural extracts on pituitary function, excluding pharmacological agents or highly modified derivatives of plant origin —such as opioids (from *Papaver somniferum*) or aspirin (from *Salix alba*)— as well as synthetic endocrine disruptors.

The vast majority of scientific evidence regarding the influence of plants on pituitary function relates to their beneficial effects, particularly in the modulation of the stress response, reproductive health and obesity via the hypothalamic–pituitary–adrenal (HPA) axis (66–70). In contrast, evidence on potentially harmful or disruptive effects—whether functional or structural—is limited and often anecdotal, highlighting a gap in current research. This imbalance is further underscored by the fact that certain plant species, such as *Ginkgo biloba* and *Garcinia kola*, have even been investigated as protective agents against known toxicants like lead acetate or sodium arsenate, owing to their antioxidant and anti-inflammatory properties (71, 72).

Several plant species have been shown to modulate the hypothalamic–pituitary–thyroid (HPT) axis. Extracts of *Lithospermum officinale*, *Lycopus virginicus*, *Melissa officinalis*, and *Thymus serpyllum* have demonstrated the ability to suppress both serum and pituitary TSH levels in animal models, even under hypothyroid conditions (73). These effects appear to combine central hormone-blocking actions with peripheral thyroid hormone-like activity. Other species such as *Chelidonium majus*, *Curcuma longa*, *Dorema aucheri*, and *Peganum harmala* contain flavonoids or alkaloids capable of modulating thyrotropin–releasing hormone (TRH) and TSH secretion via dopaminergic, serotonergic, or second messenger pathways (74). Specifically, flavonoids from *D. aucheri* and *Humulus lupulus* inhibit thyroid peroxidase and type-1 deiodinase, limiting iodine organification and peripheral T3 formation, whereas alkaloids from *C. majus* increase TRH and TSH release by blocking catechol-O-methyltransferase and enhancing calcium–phosphatidylinositol signaling in pituitary thyrotrophs. In contrast, harmaline alkaloids from *P. harmala* suppress TRH secretion through monoamine oxidase inhibition and serotonin accumulation, effects further modulated by leptin-mediated inhibition of neuropeptide Y neurons.

Regarding the hypothalamic–pituitary–gonadal (HPG) axis, traditional extracts from *Cola nitida*, *Afrormosia laxiflora*, and *Pterocarpus erinaceus* have been reported to inhibit ovulation and disrupt the estrous cycle in experimental models (75). *In vitro* studies further support these observations, showing that these plants selectively inhibit luteinizing hormone (LH) release in cultured rat pituitary cells, without affecting follicle stimulating hormone (FSH) secretion or cell viability (76). This inhibition appears to result from the formation of heterodimers between plant-derived compounds and basic glycoproteins, suggesting a non-cytotoxic, extracellular mechanism of gonadotropin modulation. Furthermore, *Li* et al. found that exposure to swainsonine (an indolizidine alkaloid) during pregnancy in mice led to significant disruption of reproductive hormone secretion (77). This effect was linked to impaired glycoprotein function in the anterior pituitary, resulting from the alteration of the glycosylation pattern of gonadotropins. Interestingly, some saponins such as those from *Quillaja saponaria* and *Gypsophila paniculata* have shown a stimulatory effect on LH secretion in pituitary cell cultures, in contrast to the inhibitory effects reported for other plant compounds (78). These triterpenoid and steroidal glycosides interact with membrane cholesterol, modifying lipid microdomains and, in some cases, inducing pore formation that facilitates Ca^2+^ influx and exocytotic release of gonadotropins in cultured pituitary cells. Notably, *G. paniculata* exhibits marked hemolytic activity whereas soybean saponins appear less lytic, suggesting structural heterogeneity within their sapogenin cores.

The effects of various plant-based formulations on prolactin secretion have been studied both in terms of increasing and suppressing its levels (79). Most of this research has focused on potentially beneficial outcomes—such as enhancing prolactin to support lactation, or reducing it in cases of hyperprolactinemia—rather than investigating possible dysregulation or adverse effects on pituitary control (80, 81). In line with this therapeutic perspective, two natural compounds derived from *Glycyrrhiza glabra* radix—18β-glycyrrhetinic acid and liquiritigenin—have shown antiproliferative and pro-apoptotic activity in prolactin-secreting pituitary adenoma cells (82, 83). These effects were mediated by distinct intracellular pathways (ROS–CaMKII–JNK/P38 activation and Ras/ERK inhibition, respectively) and were accompanied by a consistent reduction in prolactin synthesis both *in vitro* and *in vivo*, supporting their potential utility in the management of prolactin-related pituitary disorders.

Despite these observations, most data on plant-derived compounds come from experimental or preclinical settings, with considerable variability in extraction methods, active constituents, and dosing. The absence of standardized preparations and controlled human studies continues to limit the interpretation and translational relevance of these findings.

### 2.3 Kingdom *Monera*

**Figure 3:**
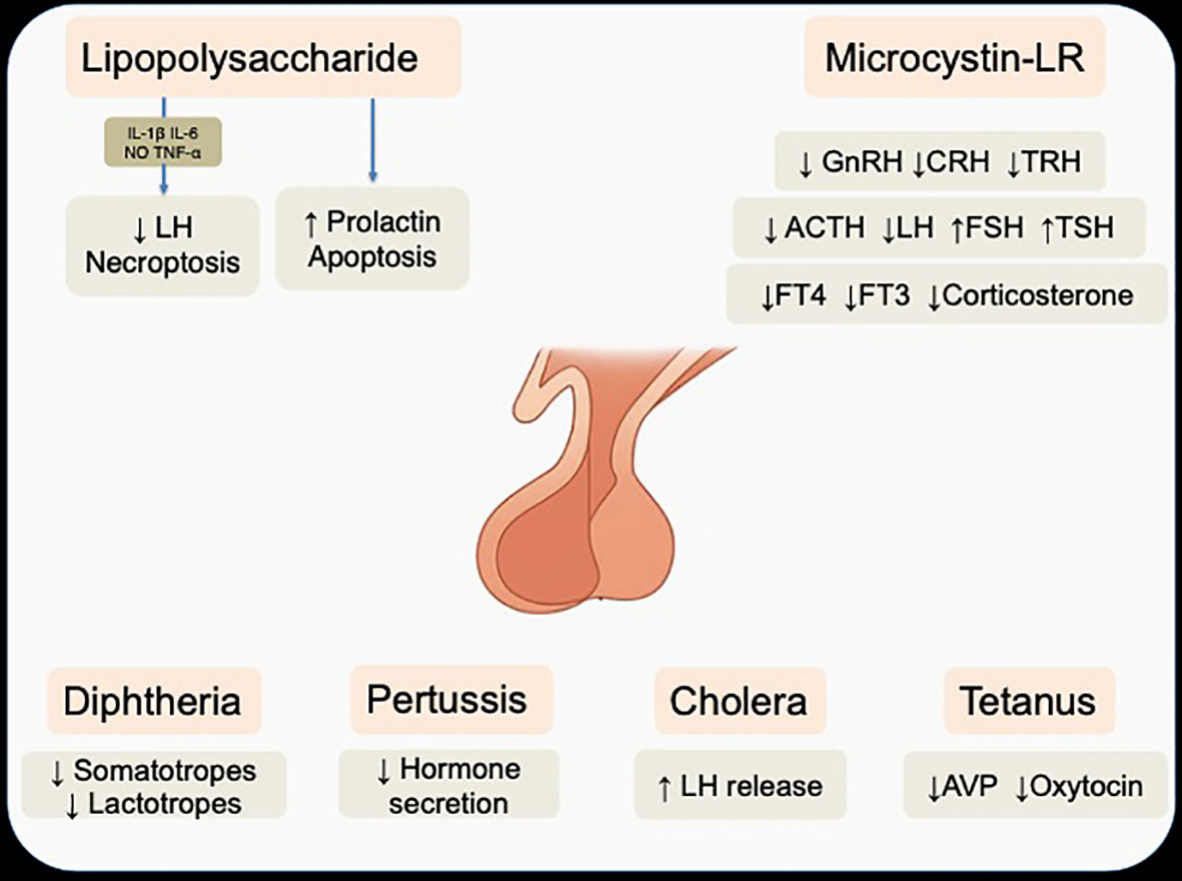
Effects of LPS, microcystin, and classical bacterial toxins on pituitary hormone regulation and cellular function. Note that lipopolysaccharide can act either directly on pituitary cells or indirectly through cytokine-mediated pathways. Acronyms: IL-1β, interleukin-1β; IL-6, interleukin-6, TNF-α, Tumor necrosis factor-α; NO, nitric oxide; GnRH, gonadotropin–releasing hormone; CRH, corticotropin–releasing hormone; TRH, thyrotropin–releasing hormone; ACTH, adrenocorticotropic hormone; LH, luteinizing hormone; FSH, follicle stimulating hormone; TSH, thyroid stimulating hormone; FT4, free T4; FT3, free T3; ADH, antidiuretic hormone. Note that the anatomical illustration of the pituitary gland was AI-generated using OpenAI DALL·E (2025) and verified by authors.

Monera comprises prokaryotic organisms characterized by the absence of a true nucleus and membrane-bound organelles (84). Beyond their well-recognized roles in infectious disease, members of this kingdom can influence endocrine function through structural components and bioactive metabolites that interact with the hypothalamic–pituitary axes without directly invading the gland, instead acting via systemic mediators. (**Figure 3**) (85) This section focuses on pituitary modulation mediated by bacterial components or metabolites and, as discussed previously, direct pituitary infections—such as abscess formation— are not included in the present analysis.

#### 2.3.1 Effect of lipopolysaccharide on pituitary function

Lipopolysaccharide (LPS) is a major structural component of the outer membrane of Gram-negative bacteria, composed of a lipid A moiety, a core oligosaccharide, and an O-antigen polysaccharide chain (86). It acts as a potent endotoxin by binding to Toll-like receptor 4 (TLR4) and triggering a strong innate immune response (87).

Evidence indicates that LPS can modulate prolactin secretion through both indirect and direct pituitary mechanisms. Regarding indirect mechanisms, LPS binds to TLR4 via LPS-binding protein and CD14, triggering the release of proinflammatory cytokines such as interleukin (IL) -1β, IL-6, and tumor necrosis factor-α (TNF-α) (88). These mediators can suppress LH secretion from gonadotropes, stimulate prolactin release via folliculostellate cell-derived IL-6, remodel the pituitary microenvironment (e.g., increased aquaporin-4 expression), and activate necroptosis in the hypothalamus, pituitary, and adrenal glands (89–92). Other mediators such as nitric oxide (NO) also participate, influencing LPS-induced prolactin release in a manner dependent on thyroid status (93). Furthermore, developmental exposure, particularly during the prenatal period, disrupts gonadotropin–releasing hormone (GnRH) neuron maturation and leads to long-term reductions in GnRH and LH and altered sex steroid profiles, whereas chronic low-dose exposure may paradoxically increase gonadotropin output through pituitary transcriptomic reprogramming (94, 95). In addition, LPS can act directly on endocrine cells, as lactotrophs express functional TLR4; stimulation promotes their proliferation and prolactin synthesis via MAPK and nuclear factor κB (NF-κB) activation—effects abolished by TLR4 blockade—demonstrating that bacterial endotoxin can influence pituitary hormonal output and cell growth independently of hypothalamic input (96). However, these direct effects have been demonstrated exclusively in experimental models, and their physiological relevance in humans with sepsis remains speculative.

On the other hand, under conditions where NF-κB–dependent survival signaling is impaired, either pharmacologically or via estradiol, LPS exposure can also trigger apoptosis in anterior pituitary cells, particularly lactotrophs and somatotrophs, accompanied by reduced expression of the anti-apoptotic protein Bcl-xL (97). Notably, LPS has also been shown to suppress the growth of TLR4-positive pituitary adenomas, an effect closely linked to IL-6 production via p38αMAPK, as evidenced by its reversal with specific kinase inhibitors (98).

#### 2.3.2 Bacterial toxins and pituitary modulation

Cyanobacteria, commonly known as “blue–green algae,” are photosynthetic prokaryotes that inhabit a wide range of aquatic environments and can form dense blooms under eutrophic conditions (99). Many cyanobacterial species produce potent secondary metabolites, including microcystins, which are cyclic heptapeptide hepatotoxins with recognized endocrine-disrupting potential (100).

In mammals, exposure to microcystin-LR (MC-LR) has been reported to reduce hypothalamic GnRH and Kiss1 transcript expression, accompanied by decreased GnRH concentrations but increased FSH levels, while effects on LH appear variable, with most studies reporting elevations, although *Dos Santos* et al. observed reduced levels (101–103).These endocrine alterations are associated with impaired gametogenesis and reduced ovarian follicle counts, indicating downstream reproductive toxicity (104). In addition to its effects on the reproductive axis, MC-LR disrupts the HPA axis by reducing hypothalamic corticotropin–releasing hormone (CRH) expression, circulating ACTH, and plasma corticosterone, and impairs the HPT axis by decreasing hypothalamic TRH expression and free T4 and T3 concentrations, while concomitantly increasing TSH levels in a compensatory manner (102, 103).

In fish and amphibians, waterborne MC-LR exposure disrupts the hypothalamic–pituitary–gonadal–liver axis, reducing GnRH, FSH*β*, LH*β* and LH receptor mRNA expression, impairing vitellogenin synthesis, and inducing gonadal dysplasia, often with sex-specific transcriptional profiles (105, 106).

Early life-stage exposures in zebrafish have demonstrated activation and dysregulation of the hypothalamic–pituitary–interrenal (HPI) axis, with an hypothalamic CRH and pituitary proopiomelanocortin (POMC) expression, elevated cortisol and altered glucocorticoid receptor expression, indicating feedback dysregulation and suggesting potential long-term programming effects (107). Combined exposures with other aquatic contaminants, such as nitrite, can exacerbate these endocrine disruptions through synergistic effects (108).

Some toxins from classical pathogenic bacteria also show pathogenic relevance for pituitary function. In rodents, the *Staphylococcus aureus* superantigen enterotoxin B and *Clostridium difficile* toxin A both increase plasma corticosterone and ACTH levels, the latter likely acting through the release of inflammatory mediators such as TNF-α and prostaglandin E_2_ (109–111). In humans, vaccination with tetanus toxoid has been associated with transient rises in cortisol and ACTH, consistent with acute HPA axis activation (112).

By contrast, although several other bacterial toxins have been investigated in relation to pituitary function, most evidence derives from animal models in which these molecules serve as experimental tools rather than as causes of clinically relevant pituitary dysfunction. For example, Diphtheria toxin has been employed in transgenic mouse models to conditionally ablate specific pituitary cell types, such as somatotropes or lactotropes, via inducible expression of the diphtheria toxin receptor (113–115). These models have revealed a remarkable regenerative capacity of the adult pituitary, involving Sox2^+^ stem/progenitor cell activation, expansion of the marginal-zone niche, proliferation of surviving endocrine cells, and, in some cases, transdifferentiation between lineages. Pertussis toxin, through ADP-ribosylation of Gi/o proteins, has been instrumental in demonstrating that somatostatin receptor signaling in pituitary cells is Gi/o-dependent, clarifying inhibitory pathways that regulate hormone secretion (116–118). Cholera toxin, in contrast, enhances GnRH-induced LH release by shifting hormone stores from a nonreleasable to a releasable pool via Gs protein activation and cyclic adenosine monophosphate (cAMP) production, a sensitization lost during desensitization but recoverable under specific stimulation patterns (119). Clostridial toxins have been used to probe vesicular trafficking and cytoskeletal regulation, for example showing that *Clostridium tetani* toxin blocks synaptobrevin-dependent exocytosis of vasopressin and oxytocin, and that other *Clostridium* toxins can induce actin depolymerization and aquaporin-2 translocation (120, 121). Collectively, these studies have provided valuable mechanistic insight into pituitary cell biology, although most represent experimental paradigms rather than evidence of clinically relevant pituitary pathology.

### 2.4 Kingdom *Fungi*

The kingdom Fungi comprises a diverse group of eukaryotic organisms that includes yeasts, molds, and mushrooms (122). Characterized by a chitinous cell wall and heterotrophic metabolism, fungi play essential ecological roles as decomposers and symbionts, but also produce a wide array of bioactive metabolites (123). Some of these secondary metabolites have been recognized as potent modulators of endocrine function, capable of affecting the hypothalamic–pituitary axes.

#### 2.4.1 Mycotoxin T-2 and pituitary impairment

Most of the current scientific evidence on pituitary dysfunction related to fungi centers around T-2 toxin, a trichothecene mycotoxin produced by *Fusarium* species (124). This compound gained notoriety in the 1940s in the former Soviet Union, where contaminated grain led to outbreaks of alimentary toxic aleukia—a severe disease characterized primarily by hematological suppression, mucosal ulceration, and high mortality (125). T-2 toxin is known to widely contaminate barley, maize, oats, and even processed human food, raising ongoing concerns about its impact on both public health and endocrine function (126).

The effects of T-2 toxin on the HPG axis appear to be highly context-dependent, varying with dose, sex, and developmental stage. In adult male mice, chronic oral exposure to T-2 toxin led to a dose-dependent decline in fertility and disrupted spermatogenesis, which correlated with reduced serum levels of GnRH, LH, FSH, testosterone, and progesterone (127). These endocrine alterations were supported by suppressed expression of hypothalamic GnRH1 and pituitary LHβ mRNA. In contrast, prepubertal exposure in female rats resulted in the opposite pattern: accelerated pubertal onset, increased circulating LH, FSH, and estradiol, and upregulation of GnRH and GnRHR expression, along with histological signs of premature reproductive maturation (128). These divergent effects suggest that T-2 toxin may suppress or activate the HPG axis depending on the physiological window, possibly through differential modulation of upstream regulators such as kisspeptin signaling.

Regarding the somatotropic axis, T-2 toxin significantly reduces GH synthesis and secretion in GH3 pituitary cells, primarily through excessive NO production via inducible NO synthase (129). This leads to mitochondrial dysfunction, oxidative stress, and caspase-mediated apoptosis, further enhanced by proinflammatory cytokines such as IL-6, IL-1β, and IL-11. These findings align with the well-established cytotoxic role of NO in the pituitary, contributing to both cell death and hormonal deficiencies (130, 131).

Beyond its effects on hormone secretion, T-2 toxin has been shown to cause direct structural damage to the pituitary gland. In fact, *Pu Guo* et al. demonstrated that T-2 crosses the blood–brain barrier and induces distinct histopathological changes in the anterior pituitary, including early signs of vascular congestion and hemorrhage, followed by apoptotic degeneration (132). The pituitary responded primarily with apoptosis, unlike other brain regions where autophagy was more prominent, suggesting tissue-specific mechanisms of toxicity.

#### 2.4.2 Other toxins and fungal molecules

Regarding other mycotoxins, zebrafish models have shown that both zearalenone (ZEA) and ochratoxin A (OTA) can disrupt pituitary-regulated endocrine function through distinct mechanisms. ZEA, a mycotoxin produced by *Fusarium* species, selectively altered LH-related pathways in adult females, as evidenced by upregulation of LHr and multiple genes involved in estrogen synthesis and steroidogenesis, while FSH receptor expression remained unchanged—suggesting a targeted effect on LH-mediated signaling (133). In contrast, OTA—produced mainly by *Aspergillus* and *Penicillium* species—did not affect prolactin release itself but interfered with its downstream action by upregulating prolactin receptor expression following miR-731 suppression in embryonic zebrafish, contributing to vascular instability (134). In addition, the mycotoxin deoxynivalenol, produced by *Fusarium* species and commonly found in contaminated cereals, has been shown to activate the HPA axis in a necroptosis-dependent manner, as evidenced by the attenuation of these effects with necrostatin-1, a well-recognized necroptosis inhibitor (135).

On the other hand, species of *Amanita* are among the most dangerous wild mushrooms due to their highly toxic compounds, including amatoxins and phallotoxins, and pose a risk of accidental ingestion (136). In relation to endocrine disregulation, *Amanita* mushroom poisoning resulted in suppressed thyroid function, with reduced thyroxine levels and undetectable triiodothyronine accompanied by inappropriately low or normal TSH concentrations in most cases, suggesting impaired hypothalamic–pituitary responsiveness or a euthyroid sick syndrome (137). Whether *Amanita* exerts a direct cytotoxic effect on pituitary cells remains unknown; this contrasts with other endocrine organs, such as the pancreas, where β-cell injury has been reported (138).

Beyond classical mycotoxins, other fungal-derived compounds may also modulate hypothalamic–pituitary function. For instance, β-glucan—a polysaccharide present in fungal cell walls—has been shown to stimulate prolactin secretion both *in vivo*, after intravenous administration, and *ex vivo* in incubated pituitary tissue (139).

Pectin derivatives may exert similar effects, suggesting that certain structural polysaccharides can influence anterior pituitary hormone release.

From a mechanistic standpoint, findings from different fungal species converge on a limited set of cellular processes—oxidative stress, inhibition of protein synthesis, and apoptotic signaling—suggesting a conserved pattern of endocrine cell injury related to redox imbalance and mitochondrial dysfunction. Such mechanisms parallel those observed in bacterial endotoxins and other biologically derived compounds, pointing to a broader susceptibility of hormone-secreting cells to metabolic and oxidative stress. Nevertheless, most available data originate from *in vitro* or rodent experiments, often employing supraphysiological concentrations, and human evidence remains largely inferential, based on dietary or environmental exposure rather than direct clinical observation.

### 2.5 Kingdom *Protista*

Protists are a diverse group of eukaryotic microorganisms traditionally defined by exclusion: they are not animals, plants, or fungi, yet share certain cellular features with each. Most are unicellular, though some form simple multicellular or colonial structures. They exhibit a wide range of nutritional strategies—including autotrophy, heterotrophy, and mixotrophy—and possess various forms of motility such as flagella, cilia, or pseudopodia (140). Despite their diversity, in practical terms the kingdom Protista is often represented by two major categories of medical and ecological interest: marine microalgae and pathogenic parasites.

#### 2.5.1 Marine microalgae

Scientific evidence is limited in this group and derives almost exclusively from two species: *Gambierdiscus toxicus* and *Prymnesium patelliferum*. In more recent taxonomic systems these species are reclassified under the kingdom *Chromista*. *Gambierdiscus* is typically placed within the *Alveolata* group, while *Prymnesium* belongs to *Haptophyta* (141, 142).

*Gambierdiscus toxicus* is a marine dinoflagellate known as the primary source of ciguatoxin, a lipophilic neurotoxin responsible for ciguatera fish poisoning —a foodborne illness characterized by gastrointestinal, neurological, and cardiovascular disturbances following the ingestion of contaminated reef fish (143). Interestingly, another compound produced by this organism, maitotoxin, has been shown to interact directly with the pituitary gland, inducing a marked increase in intracellular calcium flux in pituitary cells, likely mediated by elevated levels of inositol trisphosphate (144). This calcium influx leads to enhanced secretion of several pituitary hormones, including GH, LH, and prolactin (145). However, the regulation of prolactin secretion appears to involve additional complexity, potentially mediated by an increase in leukotriene production (146). Notably, the secretagogue effect of maitotoxin seems to be specific to the pituitary gland, as it has been shown to inhibit parathyroid hormone secretion (147).

A similar mechanism has been observed with the ichthyotoxic flagellate *Prymnesium patelliferum*, whose toxin also enhanced calcium influx in GH4C1 pituitary cells by activating both T-type and L-type voltage-gated calcium channels, leading to elevated intracellular calcium levels and a dose-dependent increase in prolactin secretion (148).

#### 2.5.2 Pathogenic parasites

Infection with *Plasmodium* spp. has been associated with impairment of the pituitary gland. Acute malaria typically activates the HPA axis, with elevated cortisol and DHEA levels, likely triggered by TNF-α and IL-1 release following erythrocyte rupture and antigen exposure during the parasitic replication phase, which acutely stimulate both the thermoregulatory center and corticotropic pathways (149). In contrast, prolonged disease may result in selective adrenal exhaustion, reflected in declining DHEA concentrations. Transient pituitary dysfunction has also been observed in the HPT axis, with low thyroxine levels and blunted TSH responses to TRH stimulation during severe malaria, which normalize upon recovery (150). However, these alterations are most likely driven by systemic inflammation due to TNF-α and interferon gamma (IFN-γ) oversecretion and metabolic adaptation to critical illness, rather than specific peptide-mediated pituitary toxicity (151).

Several protozoan infections have been associated with pituitary dysfunction. but current evidence suggests that such alterations are not mediated by selective distant pituitary modulators. In *Trypanosoma* spp. infections, extensive data from both animal models and human studies reveal central endocrine disturbances, such as ACTH exhaustion and corticotroph remodeling in *T. brucei brucei*, persistent hypogonadism in *T. rhodesiense*, and central hypothyroidism in *T. evansi* (152–154). Findings in *Trypanosoma congolense* infections are less consistent, with conflicting reports regarding ACTH dynamics, although the detection of parasites in both cerebrospinal fluid and pituitary microvasculature suggests direct involvement of the gland (155–157). Similarly, other protozoa such as *Toxoplasma gondii* and *Leishmania* spp. have been investigated for their effects on pituitary axes, particularly the HPG axis (158). Chronic *T. gondii* infection triggers sustained systemic inflammation, with IL-1 and TNF-α reaching the hypothalamus and stimulating corticotropin-releasing hormone (CRH) secretion, thereby activating the HPA axis and secondarily suppressing the HPG axis through inhibition of GnRH release. Similarly, elevated TNF-α and IFN-γ levels reported in visceral leishmaniasis may further impair hypothalamic and gonadal function, providing a plausible immuno-endocrine mechanism linking protozoan infection to reproductive axis dysfunction. Yet, no pituitary-specific toxins have been identified, and endocrine manifestations in these infections are most likely secondary to systemic cytokine-driven inflammation, though limited evidence suggests that direct pituitary involvement may occasionally occur (159–162).

Taken together, evidence from parasitic and non-parasitic protists points to two predominant processes of endocrine disruption: (1) cytokine-driven activation of hypothalamic–pituitary pathways during systemic infection, and (2) calcium-dependent exocytotic or cytopathic effects induced by marine metabolites. These phenomena illustrate how distinct protistan lineages can trigger comparable neuroendocrine alterations through immune signaling and intracellular calcium dynamics. However, most of these data derive from experimental systems rather than human studies, and the direct endocrine relevance of these mechanisms in clinical settings remains to be confirmed.

After reviewing the different biological kingdoms, the main biologically derived agents and their effects on pituitary function can be summarized comparatively. **Table 1** provides an integrative overview of representative toxins, their principal mechanisms, and documented endocrine outcomes according to the available type of evidence.

**Table 1:** Representative natural toxins affecting pituitary function, summarizing their main mechanisms, endocrine outcomes, and supporting evidence.

Kingdom	Representative agent	Main mechanism	Outcome	Evidence type
Animalia	Russell’s viper venom (metalloproteinases, serine proteases, phospholipase A_2_…)	Endothelial injury, coagulopathy	Acute and chronic hypopituitarism	Clinical and Pathological
Plantae	Swainsonine	Inhibition of N-glycan processing in glycoprotein hormones	Disruption of LH/FSH secretion and reproductive dysfunction	Experimental (*in vivo*)
Fungi	T-2 toxin	Nitric oxide–mediated apoptosis, oxidative stress	Growth hormone, gonadotropin deficiency (male mice) and citotoxic effect	Experimental (*in vivo* and *in vitro*)
Monera	LPS	TLR4 activation, cytokine release, NF-κB signaling	Altered PRL, ACTH, and HPA axis activity	Experimental (*in vivo* and *in vitro*)
Protista	Maitotoxin	Calcium influx and IP_3_-mediated exocytosis	Increased GH, LH, and PRL secretion	Experimental (*in vitro*)

## 3 Conclusions

Across the five biological kingdoms, diverse natural agents demonstrate the pituitary’s vulnerability to toxic injury. Russell’s viper envenomation is a well-documented cause of both acute and chronic hypopituitarism, while other animals can also alter pituitary responses. Plants are mainly studied for their therapeutic potential, yet certain extracts interfere with gonadotropin secretion and reproductive regulation. Fungal metabolites, particularly the trichothecene T-2 toxin, exert direct cytotoxic damage on pituitary cells, raising concerns about dietary exposure. Among kingdom *Monera*, LPS can disrupt pituitary function both indirectly through systemic inflammation and directly at the cellular level, also highlighting the influence of bacterial toxins. Protists complete this spectrum, with marine microalgae producing potent secretagogues of pituitary hormones, while pathogenic protozoa impair endocrine axes largely through systemic inflammatory mechanisms. Together, these findings underscore the pituitary’s remarkable impairment to biologically derived agents and the need for greater clinical awareness of these impactful toxic insults. Clinicians should maintain a high index of suspicion for pituitary dysfunction in acute toxic exposures, as early assessment of ACTH, cortisol, and TSH levels may unmask evolving hypopituitarism and guide timely management.
